# USE OF CHRONIC CARE MANAGEMENT SERVICE AMONG MEDICARE BENEFICIARIES IN 2015-2019

**DOI:** 10.1093/geroni/igad104.3777

**Published:** 2023-12-21

**Authors:** Jieun Jang, Dae Hyun Kim

**Affiliations:** Hebrew Senior Life, Boston, Massachusetts, United States; Harvard Medical School, Boston, Massachusetts, United States

## Abstract

The Centers for Medicare and Medicaid Services (CMS) introduced Chronic Care Management (CCM) services in 2015 for patients with multiple chronic diseases. Few studies examined the use of CCM services by demographic, geographic, and clinical characteristics. We used 2014-2019 Medicare claims data from a 5% random sample of fee-for-service beneficiaries aged 65 years or over and enrolled Medicare Part B. Of the approximately 1.4 million beneficiaries annually, approximately 75% were potentially eligible beneficiaries of CCM services who had been diagnosed with two or more chronic conditions before the year (2015: 1,073,729; 2016: 1,094,277; 2017: 1,106,929; 2018: 1,118,386; 2019: 1,130,523). The primary outcome was the rate of patients receiving CCM services among potentially eligible beneficiaries using CPT codes 99490, 99491, and 99487 in each year. Among the potentially eligible beneficiaries, the rate of patients receiving CCM services increased from 1.1% in 2015 to 3.4% in 2019 (2015: 11,480; 2016: 22,154; 2017: 31,890; 2018: 37,341; 2019: 38,863). CCM use was concentrated in the South (2015: Georgia: 1.4%; Vermont: 0.1%; 2019: Georgia: 6.1%; Vermont: 0.1%). The increase was higher in beneficiaries with ten or more chronic diseases than in those with two to five chronic diseases (2015: 2-5 chronic diseases: 0.7%; 10 + chronic diseases 2.0%; 2019: 2-5 chronic diseases: 2.1%; 10 chronic diseases: 7.0%), and higher in severely frail beneficiaries than in robust beneficiaries (2015: robust: 0.6%; severely frail: 3.3%; 2019: robust: 1.9%; severely frail: 9.4%). Variations were observed in the utilization of CCM services across demographic, geographic, and clinical characteristics.

